# Survival analysis of myopic regression after small incision lenticule extraction and femtosecond laser-assisted laser in situ keratomileusis for low to moderate myopia

**DOI:** 10.1186/s40662-022-00300-7

**Published:** 2022-08-01

**Authors:** Jihong Zhou, Wei Gu, Yan Gao, Wenjuan Wang, Fengju Zhang

**Affiliations:** 1 Beijing Tongren Eye Center, Beijing Tongren Hospital, Beijing Ophthalmology and Science Key Lab, Capital Medical University, No. 1 Dongjiaomin Xiang, Dongcheng District, Beijing, 100730 China; 2 Beijing Aier Intech Eye Hospital, Beijing, China

**Keywords:** SMILE, FS-LASIK, Myopic regression

## Abstract

**Background:**

To report the predictive factors of myopic regression in patients who had undergone small incision lenticular extraction (SMILE) and femtosecond laser-assisted laser in situ keratomileuses (FS-LASIK) after 3–12 months of follow-up.

**Methods:**

This retrospective case series study recruited patients with a subjective sphere of − 1.00 to − 6.00 D myopia. SMILE was performed in 1629 eyes of 1629 patients with a subjective refraction spherical equivalent (SEQ) of − 4.57 ± 1.20 D and 1414 eyes of 1414 patients with a subjective SEQ of − 4.53 ± 1.26 D in FS-LASIK. Refractive outcomes were recorded at 1 day, 1 week, and 1, 3, 6, and 12 months postoperatively. Predictors affecting myopic regression and other covariates were estimated with a Cox proportional hazard (Cox PH) model for the two surgical methods.

**Results:**

At 12 months, no significant difference was evident in the efficacy (*P* = 0.934), predictability (*P* = 0.733), or stability (*P* = 0.66) between FS-LASIK and SMILE. The survival rates were 83.7% in the FS-LASIK group and 88.1% in the SMILE group. Multivariate analysis by the Cox PH model revealed a similar probability of postoperative myopic regression with SMILE or FS-LASIK (*P* = 0.630). Predictors of myopic regression included preoperative higher-order aberration root mean square with 3 mm pupil diameter (pre-HOA-RMS_3_) (*P* = 0.004), anterior chamber depth (ACD) (*P* = 0.015), pre-subjective sphere (*P* = 0.016), corneal diameter (*P* = 0.016), optical zone (OZ) (*P* = 0.02), and predicted depth of ablation (DA) (*P* = 0.003).

**Conclusion:**

SMILE and FS-LASIK had a similar risk of myopic regression for low to moderate myopia. Pre-HOA-RMS_3_, ACD, pre-subjective sphere, corneal diameter, OZ, and predicted DA were predictors of myopic regression.

## Background

In 2003, Ratkay-Traub et al. [1] reported that femtosecond laser-assisted laser in situ keratomileuses (FS-LASIK) had many advantages over mechanical microkeratome, including fewer free caps, buttonholes, and irregular flaps. The disadvantages of FS-LASIK, by contrast, included its relatively complicated procedures involving the femtosecond laser used to make the flap and the excimer laser used to perform corneal reshaping. In 2006, Sekundo et al. [2] carried out the first clinical study of small incision lenticule extraction (SMILE). The SMILE procedure preserved more nerve fibers and corneal biomechanical strength, thereby potentially diminishing dry eye and ectasia.

Recent studies have indicated that biomechanical stability and predictability are better with SMILE than with FS-LASIK [3], although Li et al. [4] found no statistically significant difference. Myopic regression following corneal laser refractive surgery has recently become a concern. Moshirfar et al. [5] showed that the epithelial and stromal responses might underlie the mechanisms of optical regression. Blum et al. [6] enrolled 31 patients who had undergone SMILE and found regression of − 0.35 ± 0.66 diopters (D) over a ten-year follow-up.

SMILE and FS-LASIK have provided efficacy and safety for correcting myopia and myopic astigmatism in previous studies. Some previous studies [7] reported a similar efficiency and stability for both procedures, whereas others showed a difference. For example, after 3 months, Ganesh et al. [8] reported better refractive accuracy results for SMILE than FS-LASIK. By contrast, Li et al. [4] compared the spherical equivalent in SMILE and FS-LASIK groups but found no statistically significant difference over five years.

Here, we evaluated myopic regression between SMILE and FS-LASIK for − 1.00 to − 6.00 D myopic correction over a 12-month follow-up. Our overall goal was to use the Cox PH model to predict the factors that lead to myopic regression and estimate myopic regression's probability before the surgery.

## Methods

We retrospectively analyzed the case series study of subjects who underwent a myopic correction with SMILE or FS-LASIK from January 2010 to December 2019. The SMILE group included 1629 right eyes of 1629 patients, and the FS-LASIK group had 1414 right eyes of 1414 patients. All data for the study were analyzed at Beijing Aier-Intech Eye Hospital. The Ethics Committee of Beijing Aier-Intech Eye Hospital approved the project design and confirmed its adherence to the tenets of the Declaration of Helsinki. All patients were told of the risks and benefits of the surgery, and all provided informed consent.

The criteria for inclusion in the study were a subject sphere from − 1.00 to − 6.00 D, cylinder less than 6.00 D, age of at least 18 years, stable myopia for at least two years, and eyes with a Schirmer test score of more than 10 mm. All patients had removed their soft contact lenses for at least seven days. The exclusion criteria were ocular pathology other than myopia based on Chinese refractive surgery experts’ consensus, keratoconus, suspicious corneal topography, or autoimmune disease.

### Preoperative examination and postoperative evaluations

Preoperatively, every patient underwent a standard ophthalmologic exam that included measurements of uncorrected distance visual acuity (UDVA), corrected distance visual acuity (CDVA), intraocular pressure (IOP) with noncontact tonometry (Nidek NT-510; Japan), subjective and cycloplegic refraction, slit-lamp, and retinal tests. Corneal topography, maximum K power (Kmax), minimum K power (Kmin), cylinder axis, HOARMS_3_, and dynamic pupil graphs were determined using an OPD-Scan III (NIDEK, Technologies, Gamagori, Japan). Corneal diameter (width to width), center corneal thickness (CCT), and anterior chamber depth (ACD) were measured with a Pentacam (OCULUS Wetzlar, Germany). The information on the optical zone (OZ) diameter and maximum predicted depth of ablation (DA) was extracted intraoperatively.

Follow-up visits at 1 and 7 days and 1, 3, 6, and 12 months were scheduled regularly. Postoperatively, refractions were recorded using an automatic refractometer (NIDEK ARK-510, Japan). The topographies, wavefront aberration, UDVA, and CDVA were recorded during the follow-up visits.

### Surgical technique

In this study, every surgical procedure was performed by the same skilled surgeon (JHZ). SMILE was performed with a VisuMax femtosecond system (500 kHz, Carl Zeiss Meditec AG, Jena, Germany). We applied benoxinate hydrochloride 0.4%, a topical anesthetic, two or three times preoperatively to the conjunctival sac of the eye. The laser energy was set from 105 to 135 nJ. Treatment was based on the patient’s subjective refraction centered on the corneal vertex. The small curved interface cone was used in all cases. The cap and lenticular spot-track-distance were 4.5 μm, and the cap side and lenticular side spot-track-distance were 1.8 μm. The cap thickness was designed from 100 to 130 μm, with an intended diameter of 7.1 to 7.8 mm. The OZ diameter was set at 6.0 to 6.7 mm, depending on the preoperative corneal thickness, pupil size, and the refractive error to be corrected. A small incision was created at 10 o’clock with a 2 to 4 mm side cut. The lenticule was gently separated with a spatula and extracted with a pair of forceps.

The FS-LASIK procedure was performed using the FS200 (WaveLight^®^ Alcon Surgical, Fort Worth, TX, USA) femtosecond laser to create the flap (105 to 110 μm thickness, 8.5 to 8.7 mm diameter, and a 40-degree superior hinge), followed by EX-500 excimer laser with wavefront-optimized (WFO) ablation. The diameter of the OZ employed was 6.00 to 6.70 mm. The corneal flap was rinsed with a saline solution and restored in the stromal bed after the ablation.

Patients were informed about emmetropia and presbyopia, and a monovision strategy was used for those around 40 years old. The maximum intended postoperative refraction was − 0.25 to − 0.50 D when the patient chose monovision. All patients received steroidal and antibiotic eye drops [e.g., 0.1% fluorometholone and 0.3% ofloxacin (Santen, Japan)] four times per day for one week. We suggested using artificial tears four times a day for 3 months postoperatively.

Myopic regression was defined as residual myopia of less than − 0.50 D and a shift toward myopia of more than 0.50 D during at least 3 months of follow-up visits. The eyes were otherwise defined as not having a myopic regression [9].

### Statistical analysis

Baseline clinical characteristics are shown in Table 1. Independent sample t-tests were implemented to compare the mean ± SD in the SMILE and FS-LASIK groups. Pearson’s chi-squared test was used to analyze variables signified by frequency and percentage. The variations between SMILE and FS-LASIK at 12 months were assessed with the generalized linear mixed model. Data were analyzed using IBM SPSS^®^ ver. 23.0 for Windows (Armonk, NY, USA). The data for efficacy, predictability, safety, and stability were plotted using Microsoft Excel.

**Table 1:** Baseline demographic and preoperative characteristics of study eyes in FS-LASIK and SMILE for (− 1.00 to − 6.00 D) myopia

Variable	FS-LASIK (n = 1414)	SMILE (n = 1629)	*P* value
Sex, n (%)			0.399
Male	576 (40.7)	638 (39.2)	
Female	838 (59.3)	991 (60.8)	
Mean age (years)	27.49 ± 6.78	28.38 ± 6.91	0.000
With contact lenes, n (%)	861 (60.9)	946 (58.1)	0.114
Pre-UCVA	0.14 ± 0.10	0.13 ± 0.10	0.016
Pre-CDVA	1.00 ± 0.06	1.00 ± 0.05	0.227
Flap/cap thickness (μm)	107.69 ± 4.65	120.14 ± 5.03	0.000
Mean manifest refraction			
Sphere (D)	− 4.17 ± 1.24	− 4.19 ± 1.17	0.710
Cylinder (D)	− 0.72 ± 0.70	− 0.76 ± 0.67	0.124
Axis (^o^)	76.26 ± 71.88	73.62 ± 72.30	0.315
SE (D)	− 4.67 ± 1.18	− 4.63 ± 1.17	0.694
Pre-IOP (mmHg)	15.49 ± 2.60	15.55 ± 2.61	0.517
Cornea diameter (mm)	11.31 ± 0.44	11.59 ± 0.39	0.000
Pre-CCT (μm)	536.32 ± 30.93	537.27 ± 28.87	0.380
Pre-HOARMS3	0.26 ± 0.12	0.25 ± 0.13	0.005
ACD (mm)	3.22 ± 0.27	3.16 ± 0.27	0.000
Depth of ablation (μm)	72.40 ± 18.65	96.48 ± 17.84	0.000
Optical zone (mm)	6.38 ± 0.25	6.49 ± 0.09	0.000

*SMILE* = small incision lenticule extraction group; *FS-LASIK* = femtosecond laser-assisted laser in situ keratomileusis group; *pre-UCVA* = preoperative uncorrected distance visual acuity; *pre-CDVA* = preoperative corrected distance visual acuity; *pre-IOP* = preoperative intraocular pressure; *pre-CCT* = preoperative central corneal thickness; *pre-HOARMS3 *= preoperative high-order aberration of root mean square for 3 mm pupil size; *ACD* = anterior chamber depth; Data are presented as mean ± SD

The cumulative survival rate was analyzed by a log-rank test and plotted based on the Kaplan-Meier curves for the SMILE and FS-LASIK groups. The Cox PH model was used to correct the deviation after determining the significant variables in the univariate analysis and examining the impact of variables on the odds of myopic regression. This model can be expressed as follows:

$$H\left(t\right)/{H}_{0}\left(t\right)=e\left(\beta 1X1+\beta 2X2+\dots \beta iXi\right),$$

where *H(t)/H*_*0*_*(t)* is the hazard ratio (HR) that indicates the probability of events happening in time *t* and *X* describes the covariates (e.g., age, subjective sphere, K value, etc.) Statistical significance is defined as *P* < 0.05.

## Results

### Baseline characteristics

Table 1 shows the preoperative characteristics and intraoperative parameters in the FS-LASIK and SMILE groups for (− 1.00 to − 6.00 D) myopia.

### Efficacy and safety

**Fig. 1:**
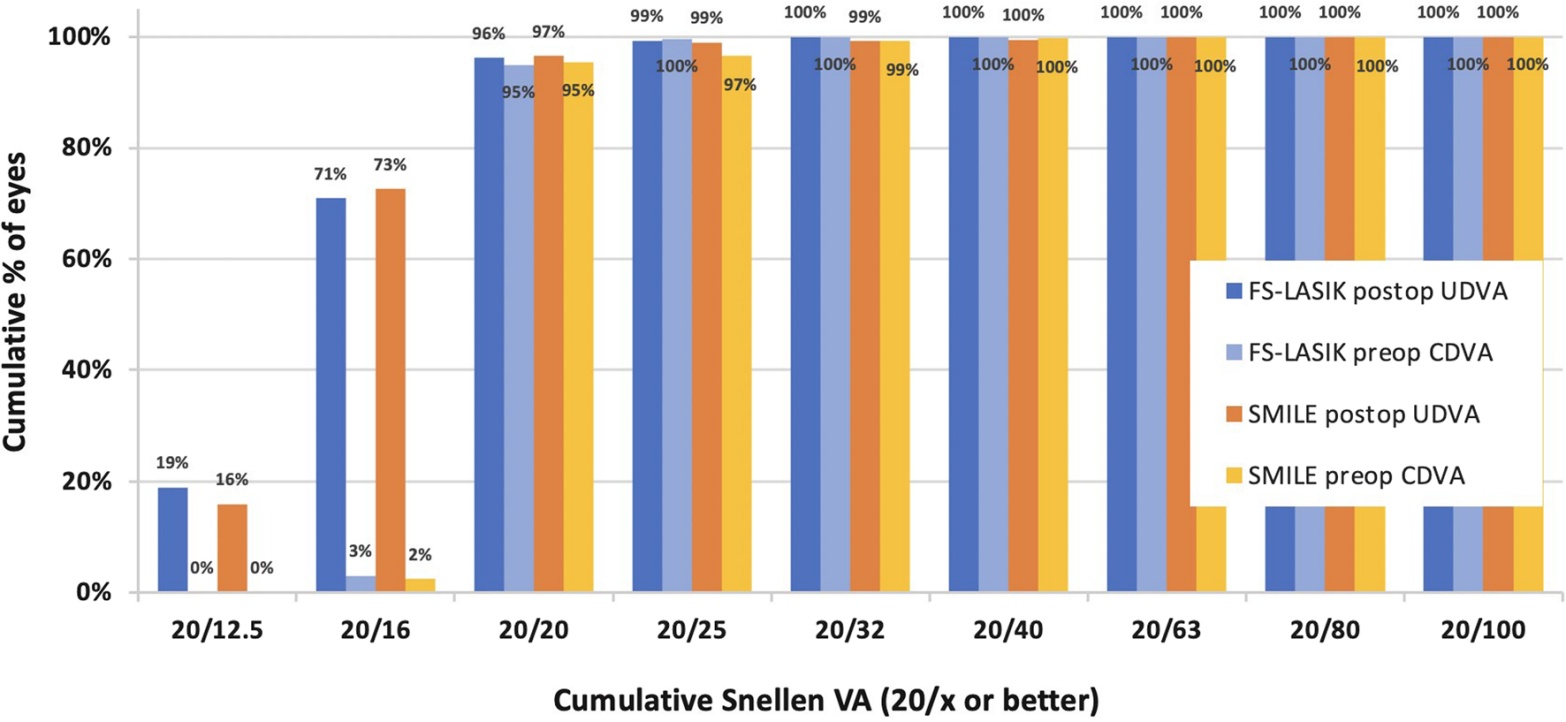
Uncorrected distance visual acuity (UDVA) at 12 months after SMILE and FS-LASIK. *SMILE,* small incision lenticule extraction; *FS-LASIK,* femtosecond laser-assisted laser in situ keratomileusis; *CDVA*, corrected distance visual acuity

At the 1-year follow-up, the mean UDVA improved to − 0.07 ± 0.07 logMAR (range 0.30 to − 0.18 logMAR) in the SMILE group and to − 0.07 ± 0.07 logMAR (range, 0.22 to − 0.18 logMAR) in the FS-LASIK group (*P* = 0.934). Figure 1 shows the cumulative Snellen visual acuities. The CDVA was − 0.07 ± 0.06 logMAR (range, 0.10 to − 0.18 logMAR) in the SMILE group and − 0.07 ± 0.06 logMAR (range, 0.10 to − 0.18 logMAR) in the FS-LASIK group. At the 1-year follow-up, 296 right eyes of the FS-LASIK group and 409 right eyes of the SMILE group had not lost one or more lines of CDVA in either of the two groups.

### Predictability

**Fig. 2:**
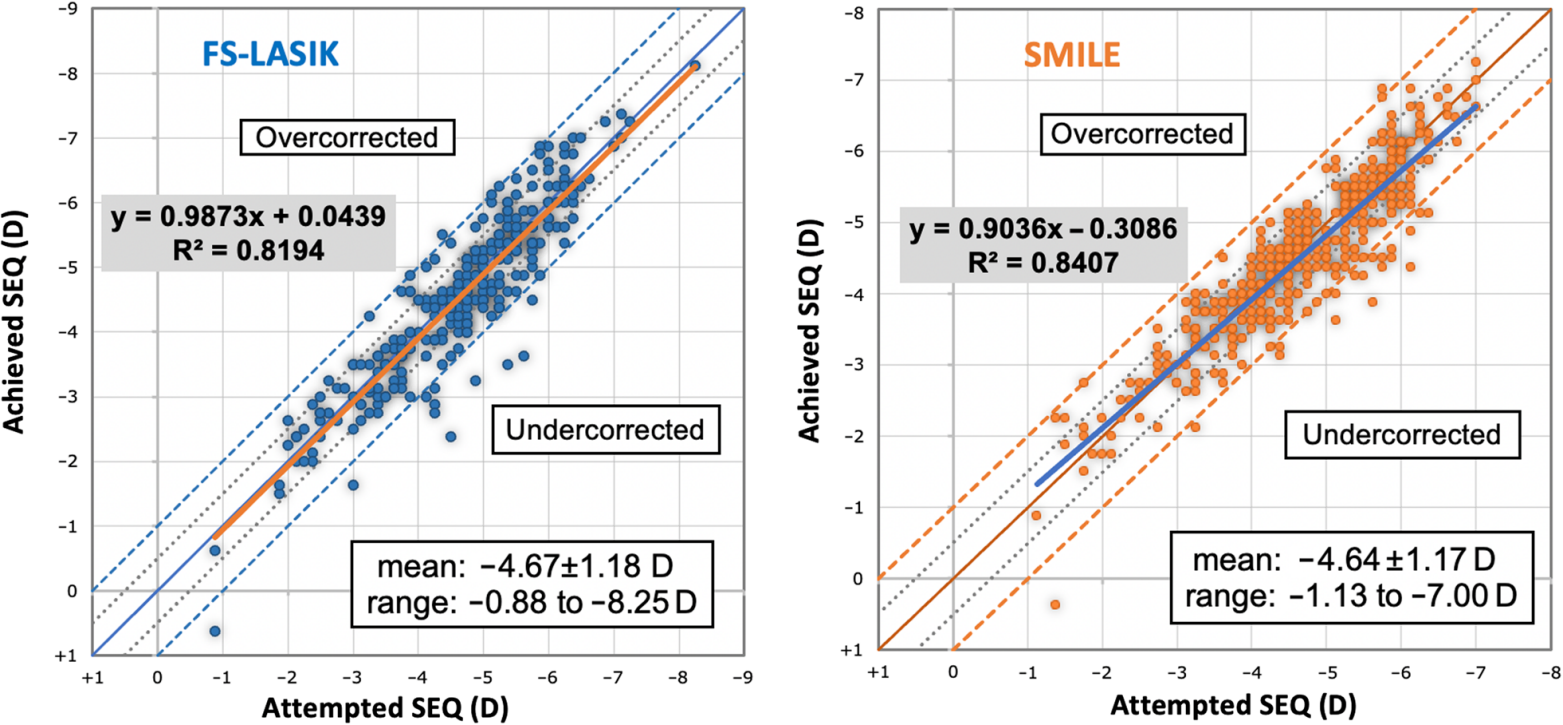
Spherical equivalent (SEQ) refraction attempted vs*.* achieved at 12 months after SMILE and FS-LASIK. *SMILE,* small incision lenticule extraction *FS-LASIK,* femtosecond laser-assisted laser in situ keratomileusis

**Fig. 3:**
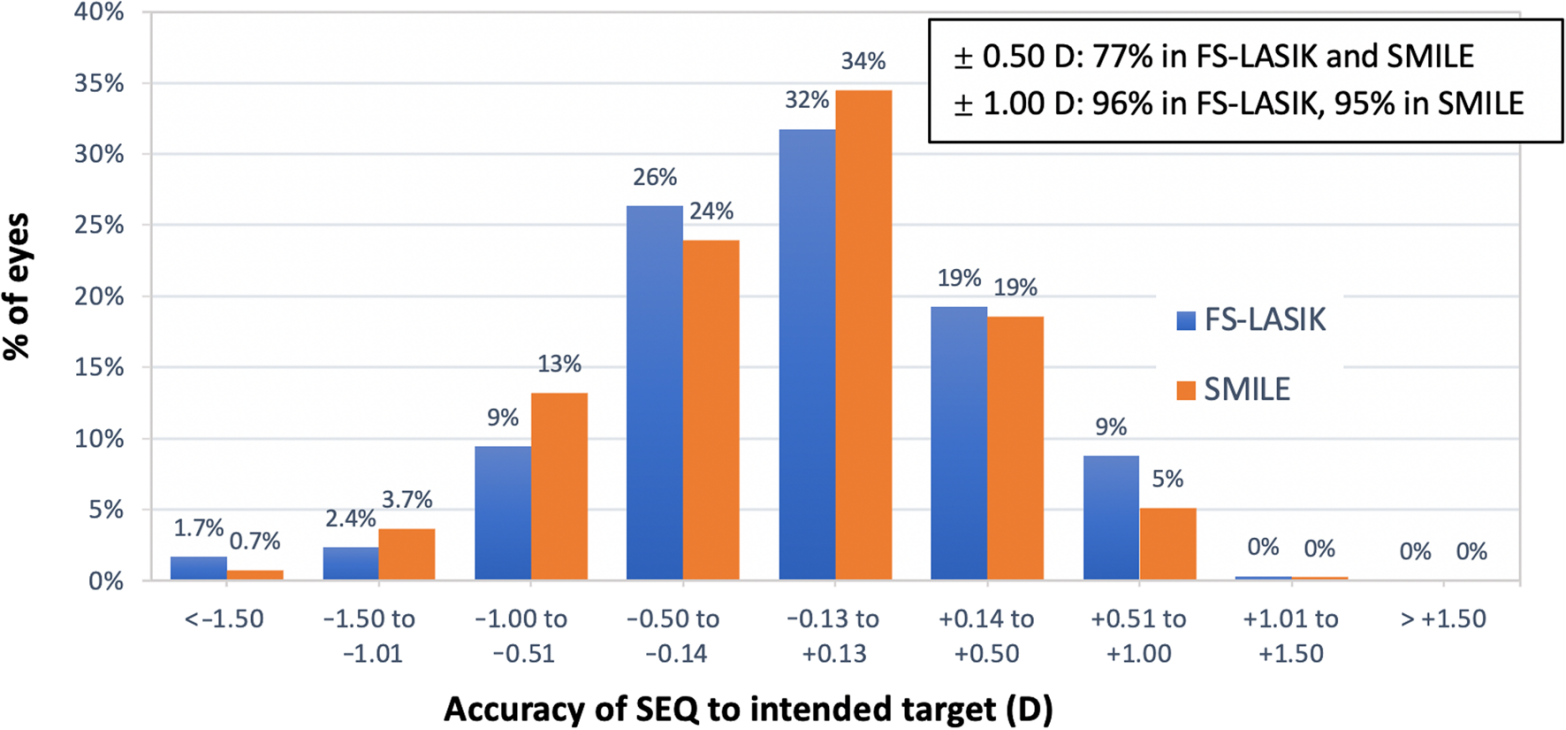
Postoperative spherical equivalent (SEQ) refraction accuracy at 12 months: percentage of eyes within ± 0.50 D and ± 1.00 D of emmetropia. *SMILE,* small incision lenticule extraction; *FS-LASIK,* femtosecond laser-assisted laser in situ keratomileusis

Figure 2 shows the attempted versus the achieved spherical equivalent (SEQ), depicted as a scatter plot. Postoperatively, eyes that were within ± 0.50 D accounted for 77.02% and 77.36% of eyes in the SMILE and FS-LASIK groups, respectively (*P* = 0.954). Eyes within ± 1.00 D accounted for 95% and 96% of the eyes in the SMILE and the FS-LASIK groups, respectively (*P* = 0.733; Fig. 3).

### Stability

**Fig. 4:**
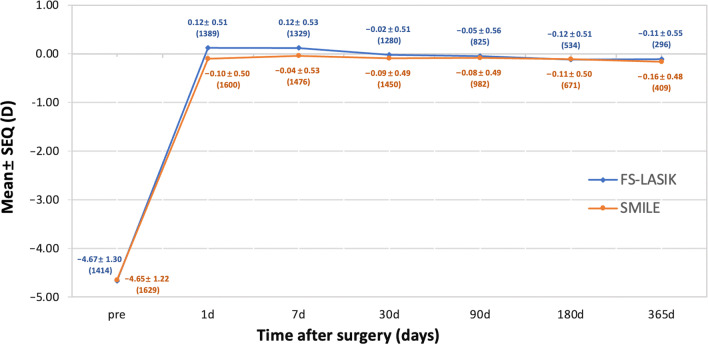
Spherical equivalent (SEQ) refraction stability with time-dependent changes 12 months after SMILE and FS-LASIK. *SMILE,* small incision lenticule extraction; *FS-LASIK,* femtosecond laser-assisted laser in situ keratomileusis

The mean postoperative SEQ values were − 0.09 ± 0.49 D, − 0.08 ± 0.49 D, − 0.11 ± 0.50 D, − 0.15 ± 0.47 D for the SMILE group and − 0.02 ± 0.51 D, − 0.05 ± 0.56 D, − 0.12 ± 0.51 D, − 0.10 ± 0.55 D for the FS-LASIK group at 1, 3, 6, and 12 months, respectively. Myopic regressions of − 0.059 ± 0.60 D were observed in the FS-LASIK group and − 0.077 ± 0.58 D in the SMILE group. From 3 to 12 months, no statistically significant difference was noted in the FS-LASIK group (*P* = 0.098), whereas significant diversity was observed in the SMILE group (*P* = 0.008). At 12 months, SEQ showed comparable outcomes in the FS-LASIK and SMILE groups (*P* = 0.66; Fig. 4).

### The cumulative survival rate and predictors of myopic regression

**Fig. 5:**
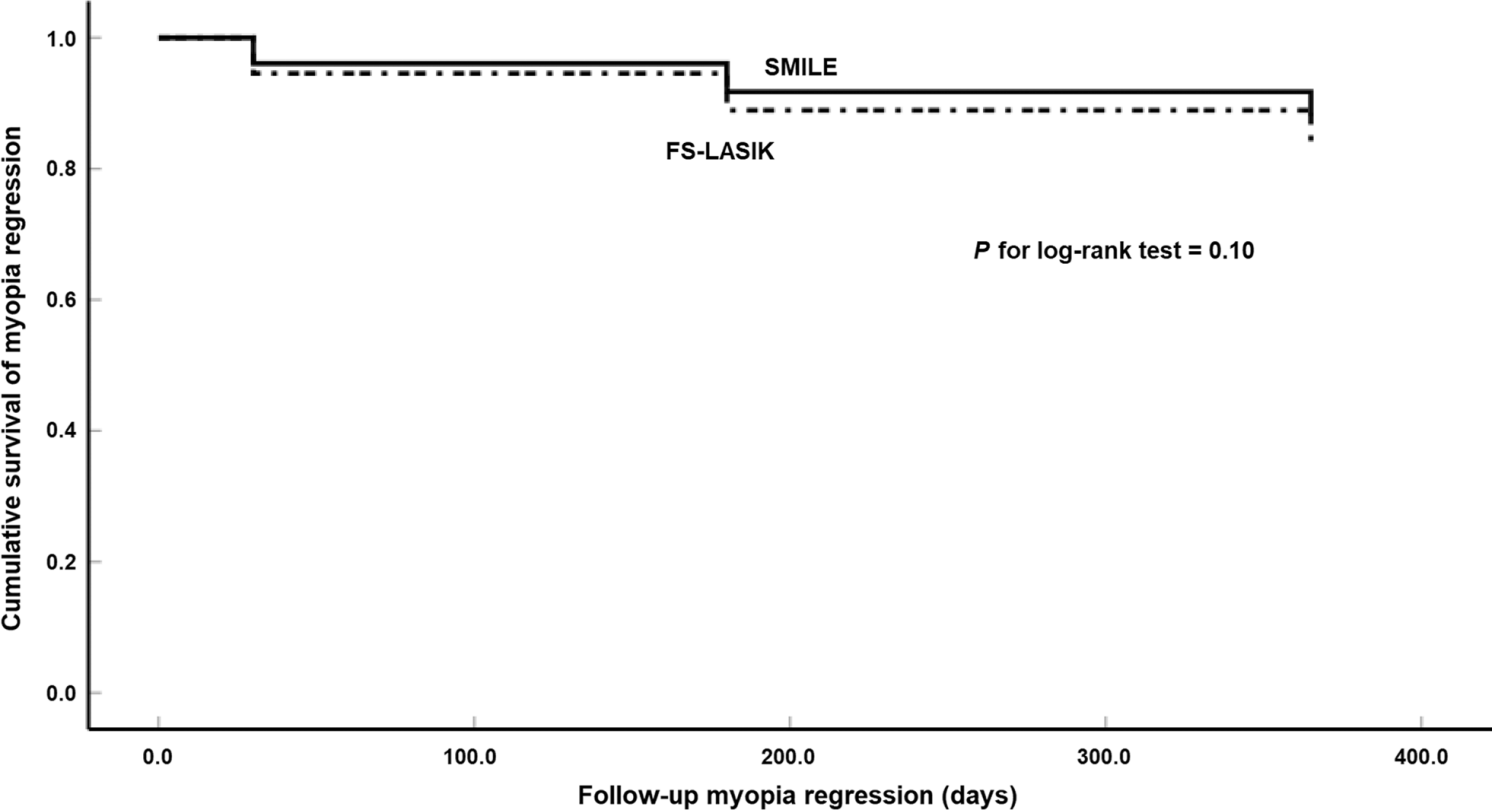
Kaplan-Meier curves of risk for myopic regression comparing FS-LASIK and SMILE using the log-rank test. *SMILE,* small incision lenticule extraction; *FS-LASIK,* femtosecond laser-assisted laser in situ keratomileusis

**Fig. 6:**
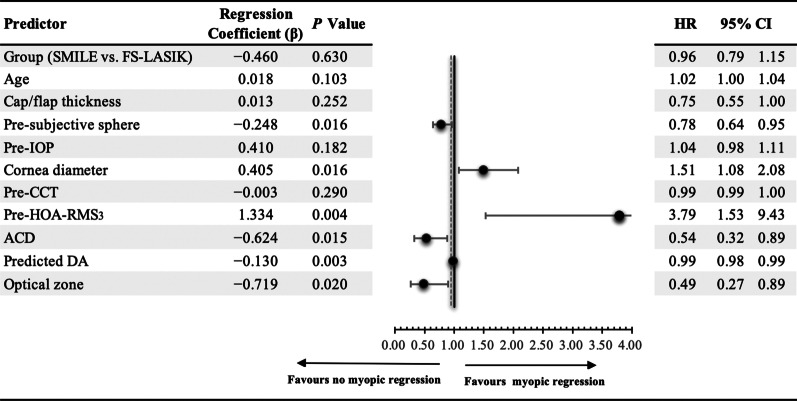
Forest plot of hazard ratios for myopic regression in the Cox proportional hazards model at 12 months for − 1.00 to − 6.00 D (n = 3043 right eyes). *SMILE,* small incision lenticule extraction; *FS-LASIK,* femtosecond laser-assisted laser in situ keratomileusis; *IOP,* intraocular pressure; *pre-CCT,* preoperative central corneal thickness; *pre-HOA-RMS3,* preoperative high-order aberration root mean square for 3 mm pupil size; *ACD,* anterior chamber depth; *DA,* depth of ablation; *HR,* hazard ratio; *CI,* confidence interval

The cumulative survival rates at 12 months were 88.1% in the SMILE group and 83.7% in the FS-LASIK group. Univariate analysis of the log-rank test and the Kaplan-Meier curves showed (Fig. 5) that the risk of postoperative myopic regression was higher for the FS-LASIK group than for the SMILE group (*χ*^*2*^ = 6.657, *P* = 0.010), but further multivariate analysis with the Cox PH model to ascertain the analogous risk of myopic regression showed non-significance (*P* = 0.63) between the two methods. Subsequent rectification of the other covariates in the 12 months of follow-up identified preoperative higher-order aberration root mean square with 3 mm pupil diameter (pre-HOA-RMS_3_), corneal diameter, ACD, pre-sphere, OZ, and predicted DA as significant predictors. Age, the thickness of the flap or cap, pre-IOP, and pre-CCT were not significant predictors of myopic regression (see Fig. 6).

## Discussion

SMILE is a comparatively new method that has only been applied in clinical practice since 2011 [10]. Still, it is recognized for its superiority in achieving better corneal biomechanical stability [3]. In this study, SMILE and FS-LASIK revealed no statistical differences in terms of their efficacy, safety, predictability, or stability for myopic corrections of − 1.00 to − 6.00 D, in agreement with previous studies [7, 11]. Our results also revealed that SMILE and FS-LASIK had the same probability of myopic regression and ascertained predictors affecting myopic regression. To the best of our knowledge, this study is the first to investigate myopic regression in a large number of patients with myopia from − 1.00 to − 6.00 D and the first to discuss the relevant predictors of myopic regression for the SMILE and the FS-LASIK procedures.

### Variation in myopic regression

The mean postoperative SEQ was − 0.15 D in the SMILE group and − 0.10 D in the FS-LASIK group at 12 months. This finding was similar to previously published results that indicated a tendency toward a more myopic residual SEQ, at − 0.01 to − 0.33 D in SMILE versus − 0.02 to − 0.17 D in FS-LASIK [12]. Overall, 77.02% of the eyes in the SMILE group and 77.36% in the FS-LASIK had a postoperative SEQ within ± 0.50 D, which is the approximate value published in a previous study [13]. In total, 95% of the eyes in the SMILE group and 96% in the FS-LASIK group were within ± 1.00 D of the targeted SEQ, agreeing with previous reports [13, 14].

We also provide the first evaluation and comparison of the rates of myopic regression for a SMILE and an FS-LASIK group. The incidence of myopic regression was lower in our FS-LASIK group than in a previous study by Lin et al. [9], who showed that 43.6% of eyes that underwent FS-LASIK had myopic regression. The higher myopic regression rate might be attributed to eyes with more severe myopia within − 10.00 D in their study compared with ours (within − 6.00 D). Their preoperation average SEQ (− 6.17 ± 1.86 D) was more elevated than ours (− 4.51 ± 1.29 D). Myopic regression would vary according to myopic diopter if the definition and inclusion criteria were uniform. We paid attention to the myopic diopter, the definition and inclusion criteria, method of surgery, and follow-up time when we compared the percentage of myopic regression [9]. The impact was more evident within − 0.50 D and became insignificant when the definition of myopic regression increased to − 1.00 D, as in the study by Lin et al. [9].

According to our univariate analysis, the myopic regression rate was higher with FS-LASIK than with SMILE. The cumulative risk for myopic regression was 16.3% for FS-LASIK and 11.9% for SMILE at 12 months (*P* = 0.010). This result was supported by the findings of Reinstein et al. [15], who reported that the intact anterior lamella remained stronger after SMILE than after LASIK, according to the mathematical model of Randleman et al. [16, 17].

We also rectified the differences between the two groups with the Cox PH model for some variables, including the mean age of 27.49 ± 6.78 years in FS-LASIK and 28.38 ± 6.91 years in SMILE (*P* = 0.000) and the pre-HOA-RMS_3_ of 0.26 ± 0.12 μm in FS-LASIK and 0.25 ± 0.13 μm in SMILE (*P* = 0.005). No significant difference was evident for myopic regression after all covariates were adjusted. This result could be supported by the findings of Agca et al. [13], who reported that corneal hysteresis (CH) and a corneal resistance factor (CRF) did not differ significantly between SMILE and FS-LASIK postoperatively. The similarity of the biomechanical effects after SMILE and FS-LASIK might sustain this parallel occurrence of myopic regression. Kanellopoulos [18] showed that the SMILE procedure reduces the tensile strength in lower myopic corrections while retaining a similar tensile strength in higher myopic correction compared with LASIK. Magallanes et al. [19] proved that the significant loss of corneal strength following large myopic corrections might lead to refractive regression.

### Predictors for myopic regression

Factors that predicted myopic regression varied in previous studies. The mechanism of myopic regression might include corneal remodeling [5] and biomechanical changes [20] in the anterior and posterior corneal surfaces [21, 22], axial length elongation, and nuclear sclerosis by age during the long-term follow-up period [23]. The identified predictors that affected myopic regression after LASIK included a higher degree of astigmatism [24], the subjective SEQ [20], age [25], IOP [26, 27], and OZ diameter [28]. The significant factors that affected myopic regression in the present study included pre-HOA-RMS_3_, pre-subjective sphere, ACD, corneal diameter, OZ, and predicted DA.

The pre-HOA-RMS_3_ (HR = 3.79, *P* = 0.004) was a risk factor for myopic regression. In this study, pre-HOA-RMS_3_ was a 3 mm pupil diameter root mean square of high-order aberration. Previous studies found that higher HOA-RMS facilitates myopia progression [29]. High-order aberrations induced by imperfections in the cornea and lens might be inherited from the patients’ parents, and genetic contributions to myopia have been recognized in previous studies [30]. Higher HOA-RMS_3_, which degrades the retinal image, may accelerate the eye's axial elongation, and increase myopia [31]. Another reason may be that HOA-RMS_3_ increases with accommodation [32], and the refractions in the postoperative period were recorded using an automatic refractometer with accommodation. An accommodated status would be inclined to having more severe myopia. Further prospective studies are needed on the effects of HOA-RMS_3_ on myopic regression.

ACD (HR = 0.54, *P* = 0.015) was a protective factor against myopic regression. One reason might be that ACD with non-accommodation was deeper than the accommodation process [33]. The ACD became 1.5% shallower per diopter of accommodative demand, with a median of about 0.99% at − 1 D, 2.63% at − 2 D, and 4.47% at − 3 D for ACD with accommodation. Chen et al. reported that eyes with cycloplegics, non-accommodation, would increase ACD depth and decrease SEQ in low to moderate myopic eyes [34]. Therefore, the eye with accommodation tended toward greater myopia [35]. The other eyes with similar axis length would have deeper ACD and lower SEQ, according to Nakao et al. [36], and less myopic regression.

In our study, the corneal diameter (HR = 1.51, *P* = 0.016) was a risk factor for myopic regression. The corneal diameter might reflect the eyeball’s size [36], and the eyeball extension from back to front was hypothesized to be a more significant factor than equatorial ocular expansion in axial myopia, based on the mechanical tension theory [37, 38]. A more prominent cornea diameter resulted in a higher myopia SEQ. In our study, the higher pre-sphere led to more significant myopic regression.

As in previous studies, a higher pre-subjective sphere (HR = 0.78, *P* = 0.016) increased myopic regression [28, 39]. A larger OZ (HR = 0.49, *P* = 0.020) was a protective factor against myopic regression but only had a minor effect, in agreement with previous reports [28, 39]. The predicted DA (HR = 0.99, *P* = 0.003) correlated with the spherical equivalent, OZ, and ablation method (aspherical and spherical). A deeper DA decreased the myopic regression, consistent with previous study findings that aspherical ablation could conserve the DA in cases of higher myopia or a thinner cornea, and yet it might increase myopic regression more than spherical ablation [39].

Multivariate analysis with the Cox PH model, conducted to ascertain the impact factors for myopic regression, revealed the HR of myopic regression in 12 months. Using the Cox PH model, the HR of each covariate was obtained to determine the impact of each factor on the risk of myopic regression. Thus, when patients asked for a preoperative consultation, the clinicians could inform them of the possibility of myopic regression according to each patient’s situation.

Our study is not without limitations. It was more convenient to obtain postoperative refraction by autorefraction rather than subjective and cycloplegic refraction at each follow-up visit. However, considering the age of our study participants (adults), the accommodation should not have markedly affected the results. In support of this, Pesudovs [40] showed excellent agreement using subjective refraction, and this was shown to be unaffected by refractive surgery. A second limitation of this retrospective study and selecting the right eyes was the probability of selection bias that could not be excluded. Therefore, some covariates are confounding variables for estimating the myopic regression between the FS-LASIK and SMILE groups. We adjusted all the covariate differences by multivariate analyses with a Cox PH model to minimize their influence on myopic regression. We have also chosen the right eyes as previous studies show high interocular symmetry in bilateral eyes such as refraction [41], corneal biometrics [42, 43], ACD, and IOP [44]. In the future, we plan to devise a prospective and randomizing study to investigate predictors of myopic regression after laser refractive surgery.

## Conclusions

This study suggests that SMILE and FS-LASIK have analogous odds of subsequent myopic regression, as low to moderate myopia, lower subjective sphere, deeper ACD, larger OZ, and deeper ablation decreased the myopic regression. In contrast, higher HOA-RMS_3_ and a larger corneal diameter might increase myopic regression. Prior to surgery, we could identify a patient with a higher risk of myopia regression and more precisely estimate the probability using this predictive model.

## Data Availability

The datasets used and analyzed during the current study are available from the corresponding author on reasonable request.

## Abbreviations

SMILE: Small incision lenticular extraction
FS-LASIK: Femtosecond laser-assisted laser in situ keratomileuses
SEQ: Spherical equivalent
D: Diopter
VA: Visual acuity
Cox PH: Cox proportional hazard
logMAR: Logarithm of the minimal angle of resolution
Pre: Preoperative
pre-HOA-RMS_3_: Preoperative high-order aberration root mean square with 3 mm pupil diameter
ACD: Anterior chamber depth
OZ: Optical zone
DA: Depth of ablation
UDVA: Uncorrected distance visual acuity
CDVA: Corrected distance visual acuity
IOP: Intraocular pressure
CCT: Center corneal thickness
HR: Hazard ratio
